# Supplementary material for: A mathematical model for persistent post-CSD vasoconstriction
Source: PLoS Comput Biol. 2020 Jul 15;16(7):e1007996. doi: 10.1371/journal.pcbi.1007996 (PMC7416967; doi:10.1371/journal.pcbi.1007996)
Supplement: Attachment — Submitted filename: PLOS review.pdf [file pcbi.1007996.s002.pdf]

A primary goal of this paper is to develop a computational model to help explain mechanisms underlying neurovascular malfunction following cortical spreading depression. This is an interesting issue and the modeling is novel because, as the authors point out, it involves time scales not considered in previous models for CSD. However, I have several major concerns that are outlined below.

1) The authors claim in the Discussion that they "demonstrated macroscopic vascular behavior consistent with the hour-long disruption in neurovascular coupling following cortical spreading depolarization." I interpret this to mean that there is some variable corresponding to vascular behavior (say vessel diameter), which is in some constrictive state following CSD and then returns to a resting state after about one hour. I don't see where they show this. The only place in the results section where the vascular activity is described is Figure 4; however, the muscle tension does not return to a resting state after one hour. In fact, neither do the other variables.

2) The authors need to explain the actin/myosin model (5). What do the variables correspond to and what values correspond to a constrictive or resting state?

3) The authors seem to justify their results by stating that "the dynamics in this set of simulate are consistent with the observed vasoactivity of Figure 4A in Chang et al." They need to explain this claim in some detail. It is not at all obvious how the simulations in this paper correspond to what is shown in Chang et al.

4) The authors make many, many assumptions, which raise concerns. For example:

a) The mitochondrial membrane potential $\Delta\Psi_m$ is considered to be fixed. However, a large increase in mitochondrial calcium may lead to a. large change in $\Delta\Psi_m$ and this would impact all aspects of mitochondrial dynamics.
b) It is assumed that hat large quantities of calcium have entered the mitochondria during the acute phase of CSD, through the mPTP. Isn't opening of the mPTP associated with cell death?
c) The caption of Table 5 states that "Steady state free ionic calcium concentrations enforced in all of the model simulations." But the figures show changes in these concentrations.
d) I don't understand the statement: "In these simulations the stabilizing leak fluxes are calibrated in order to maintain the original unperturbed steady state."
e) The role of ATP is confusing. At one point, the authors state: "we assume that ATP production meets the needs of this system." However, ATP production is described in some detail in, for example, Sections 2.5 and 2.6. Why is the ATP production rate proportional to $J_{mit \rightarrow cyt}$? It is possible that the large increase in mitochondria calcium leads to a sharp decrease in ATP production due to changes in $\Delta\Psi_m$ described above.
