# Supplementary material for: A mathematical model for persistent post-CSD vasoconstriction
Source: PLoS Comput Biol. 2020 Jul 15;16(7):e1007996. doi: 10.1371/journal.pcbi.1007996 (PMC7416967; doi:10.1371/journal.pcbi.1007996)
Supplement: Attachment — Submitted filename: Xu_et_al_BiophysJ_28October2019.pdf [file pcbi.1007996.s003.pdf]

Review of "A mathematical model of mitochondrial calcium-phosphate dissolution as a mechanism for persistent post-CSD vasoconstriction"

By Xu S, Chang JC, Chow CC and Huang H.

Summary: This manuscript assembles a computational model to explain elevated cytosolic calcium levels and clearance in vascular smooth muscle cells (VSMCs) of the cerebral vasculature after a cortical spreading depolarization (CSD) event. This model describes calcium transport through the cellular membrane, between the cytosol and the endoplasmic reticulum, and between the cytosol and the mitochondrial matrix. This model is able to describe the hour long timescale for cytosolic calcium to return to normal after a CSD event.

Overall Comments: The authors have built a simplified model to represent the calcium levels in VSMCs after CSD and have clearly described their model in the text. They have also effectively varied parameters to try to understand the important parts of the model. However, no experimental data is shown and in none of the figures the actual rise and decay of calcium over the post CSD time period is shown. Parameters are clearly presented along with their sources which enables reproducibility however searching for the code under the username joshchang on github did not yield the code for inspection.

Specific Comments:

1) No data is represented to challenge the results of the model.
2) Make sure that code is available on github at least and it is suggested to take advantage of the code check that is an option with PLoS Comput Biol.
3) It is stated that in figure 2 the net movement of the calcium is from the mitochondria to the cytosol to the extracellular space however the net flux from mitochondria to cytosol in figure 2 is essentially zero at the resolution of the figure.
4) The simulations of figures 3 and 4 show some variability with no apparent pattern. Why is this the case if you simply have a set of ODEs modeled here?
5) The authors have made clear that local regulatory mechanisms have been omitted in this model. However in the discussion the interaction between these Ca^{2+} transients and those generated by the myogenic response or shear mediated dilation should be discussed.

Specific Minor comments

1) Figure 4 shows the clearance of Ca^{2+} in panel 1 and the release of Ca^{2+} from the mitochondria in panel 2 but does not resolve the time course of Ca^{2+} in the cytosol over the 30 minute timescale depicted.
2) Figure 5 is not really clear. Suggest one 3D view with 3 views comparing each pair of parameters (top, front and side).
3) On page 15 the authors suggest that cell-specific measurements would be beneficial for the identification of this model. Can the authors suggest what measures would have the most utility to advance this work?
4) Mitochondrial permeability transition pore opening leads to mitochondrial dysfunction and cell death. It is assumed in this model that the VSMCs recover. Is this tractable?
