# Supplementary material for: A mathematical model for persistent post-CSD vasoconstriction
Source: PLoS Comput Biol. 2020 Jul 15;16(7):e1007996. doi: 10.1371/journal.pcbi.1007996 (PMC7416967; doi:10.1371/journal.pcbi.1007996)
Supplement: Attachment — Submitted filename: ReviewerComments-CSDpaper-PLOSCompBio.pdf [file pcbi.1007996.s004.pdf]

Review of paper

A mathematical model of mitochondrial calcium-phosphate dissolution as a mechanism for persistent post-CSD vasoconstriction

The paper deals with the modelling of the cortical spreading depolarization (CSD), a relatively slow wave propagating in cortical brain tissue during migraines, strokes etc. As such it has the potential to provide a good contribution to the literature if the following points below are addressed carefully. The experimental results the model is compared to are those in Chang et al, Figure 4A.

Points to address:

1) Please provide a clear explanation of the experimental results that you are comparing against with your model. Please include Figure 4A from Chang et al, preferably early on, for the sake of clarity.
2) Section 2: Please provide a clearer explanation of the modelling modules you have introduced for the cytosol, mitochondria and the Endoplasmic Reticulum. Why have you chosen these models vs alternatives? What are the key assumptions? Also, more specifically:
    a. in Section 2.1 the variables AP, AMp, MP and the parameters K1, K2, K3 etc in the equation system (5) are not defined. Also it is not mentioned if/where the model has been developed before. I presume in Yang et al [71]?
    b. In equation (11) why do you model J_{mcu} in this way? What is the biophysical process justifying this choice?
    c. System (21): why do you choose the model by Swaminathan et al [60] vs other established IPR models?
3) It is not clear which part of the modelling is new and which has been developed in previous work. The novelty of the work should be clearly established.
4) Section 3: The comparison of the simulations with the experimental results is not convincing. In Figure 4A of Chang et al the variables plotted and tracked are mostly different from the variables of the ODE model used in the current paper. One variable that should be definitely included, or at least discussed in detail when the modelling variables are introduced, is the Pial Artery Diameter which is constricting as the CSD passes.
5) An "hour-long" vasoconstriction effect is reported but the simulations provided run only up to 30 minutes. What happens afterwards? Can you provide some pictures over the duration of an hour?
6) Figure 2: Are there perhaps more bifurcations beyond the Hopf bifurcation?
7) Figure 2: Can you explain why the Hopf bifurcation is observed in all variables at the same value of calcium?
8) Figure 2 caption: Not sure why the sentence "Fr is proportional to the constrictive force…." Is included here. If you are leaving it here please connect better to the context.
9) CSD is a wave but only ODEs are used in the model. Spatial dependence is needed for a wave to propagate. The authors should clearly explain why they have neglected spatial effects, dealing only with oscillations and not waves.
10) p.10 "Results" section. Please explain what DSP.jl is doing. Is it a bifurcation tracking module?
11) p. 11 Why do you introduce α_i? Not clear.

12) Please make sure you use **consistent and meaningful notation**. For example:
    a. $J_{mit->mit}$ does not make much sense since it is the flux from free calcium to buffers
    b. the abbreviation ER for the Endoplasmic Reticulum has been introduced early on but subsequently the full words are used.
    c. $[Ca^{2+}]_{source}$ is defined to be $[Ca^{2+}]$ from the mitochondria into the cytosol but ER also acts as a source for the cytosolic calcium. Please clarify
    d. Do not use the small parameter ε if it Is not going to be used subsequently
    e. The references are not all written in the same way – please fix
13) **Structuring:**
    a. After the Discussion there are still three sections which give a feeling of the paper not concluding. Also, the section “Assumptions limitations, and alternative mechanisms” contains information which I would expect to see much earlier in the paper, for example the sentence “Prior models distilled the biological variability in vascular smooth muscle calcium dynamics into a few most salient mechanics which we replicate in our model” should be introduced much earlier along with the assumptions of the model, what was deemed negligible etc. The salient mechanisms should be clearly stated before laying down the equations of the model.
    b. Please provide a recap of what you have done before moving to section 2.6 or section 2.7
14) Other comments:
    a. p.2 “…impairement in the form of decreased plasticity”. Note typo
    b. p.2 “ER offers a stronger but limited basin for calcium…” what do you mean by stronger? Perhaps larger?
    c. P.6 Section2.4 the word “flux” or “behavior” is missing from the first sentence after “(NCX)”. Similar omissions elsewhere to be edited, e.g. jus before equation (29)
    d. p. 12 Figure 4 caption: All concentrations are relative
    e. p.13 there is some discrepancy in the first paragraph as you say “Eqs 32 and 33 describe…” whereas in p.10 you say that it is eq (33) that dictates release from the mitochondria.
    f. p. 15 “manuscript” –>“work”
    g. The two sentences below equation (33) and before Section 2.6 need to be reconciled.
