# Supplementary material for: A mathematical model for persistent post-CSD vasoconstriction
Source: PLoS Comput Biol. 2020 Jul 15;16(7):e1007996. doi: 10.1371/journal.pcbi.1007996 (PMC7416967; doi:10.1371/journal.pcbi.1007996)
Supplement: Attachment — Submitted filename: PCOMPBIOL-D-19-01345R1 resubmission review - Reviewer 1.pdf [file pcbi.1007996.s007.pdf]

I appreciate the authors' effort to respond to my comments.

While the authors have satisfactorily addressed some of my comments, my main concerns regarding the manuscript are for the most part unresolved, and the manuscript is minimally revised.

Based on the evidence provided in the paper, I am still not convinced that focusing almost entirely on the analysis of sustained calcium oscillations is relevant to the main objective of the manuscript, i.e., *an hour-long post CSD vasoconstriction and "quantification" of the impact of calcium phosphate dissolution on the maintenance of F0F1-ATP synthase activity*, as it appears in the abstract. Does vasoconstriction necessarily mean calcium oscillations?

Simulation results indicate that the system reaches a steady oscillatory profile within a few minutes. Since the concentration of the mitochondria is assumed to be constant, this profile would expectedly persist over the entire duration of the simulation (which can be few hours, few days, or few minutes). Therefore, contrary to the authors' claim, the simulations do not necessarily show "long lasting" post CSD vasoconstriction – simulations reach their steady state (limit cycle) and would stay there indefinitely, irrespective of the simulation time (also see comment #11). This claim would have been justified if authors had shown recovery of the vessel diameter, or equivalently the cytosolic calcium level, after almost an hour to their basal, pre CSD, levels.

Overall, despite the importance and the need for mathematical modeling of the vascular response in CSD and that the authors have made a noticeable effort in providing a detailed model showing this phenomenon, from my perspective, the conclusions drawn based on the current model are largely speculative and the model and the analysis need considerable expansion.
