# Supplementary material for: A mathematical model for persistent post-CSD vasoconstriction
Source: PLoS Comput Biol. 2020 Jul 15;16(7):e1007996. doi: 10.1371/journal.pcbi.1007996 (PMC7416967; doi:10.1371/journal.pcbi.1007996)
Supplement: Attachment — Submitted filename: PLOS Comp. Bio. Review - Xu et al.docx [file pcbi.1007996.s001.docx]

The manuscript by Xu et al. provides a compartmental model of a representative vascular smooth muscle cell to investigate the hypothesis, as well as implications of mitochondrial permeability transition pore (mPTP)-driven elevations of mitochondrial calcium concentration during the acute phase of cortical spreading depression (CSD) on the long-term vasoconstriction observed during the second phase of this phenomenon. The authors modeled the interaction between 4 Ca^2+^ pools, i.e., Cytosol, ER/SR, Mitochondria and the Extracellular space, by providing relative fluxes within and across compartments, and assuming fast buffering approximation for the cytosol and ER/SR. They assumed that the mitochondria exist at “quasi-steady” equilibrium at saturation and demonstrated that the slow release of calcium from this compartment to the cytosol can potentially explain long-term sustained elevations in the cytosol and the resultant vasoconstriction during CSD.

Although mathematical modeling of the vascular response during CSD is much needed to advance our understanding of the intricacies of this multi-mechanistic phenomenon, there are several concerns related to the study of Xu et al. that the authors should address.

1) My main concern with the current study is that even though the main findings of the manuscript lies on the dynamics of calcium in the mitochondria and the calcium-phosphate dissolution/aggregation within this compartment (as is reflected in the title, abstract and discussion), the concentration of free mitochondria calcium is assumed to be constant throughout the long-term simulations, and none of the dynamics of calcium phosphates are included in the model. What is the basis of this assumption? Why did the authors opt not to provide calcium dynamics in the mitochondria to observe how long it takes for the calcium levels to reduce to physiological values?

If the model/study is specifically designed to investigate the role of mitochondria, in particular dynamics of mitochondrial calcium phosphate dissolution during CSD, it would be much more informative to provide a more detailed dynamics of this compartment (consistent with what is assumed for the other compartments of the model, such as ER/SR, Cytosol and the plasma membrane channels) and demonstrate the validity of these assumptions as well as the findings of the study. Similarly, in the abstract, the authors state *“… we quantify a possible impact of calcium phosphate dissolution on the maintenance of F0F1-ATP synthase activity*.” without providing any equations on the dynamics of proton balance across the mitochondrial inner membrane (IMM) and the ATP production for such quantification.

2) Although it is interesting to show the presence of oscillations in the cytosolic calcium concentrations during elevated levels of mitochondrial calcium, how are these oscillations (and the analysis of the effect of different fluxes on their sustainment) relevant to the main objective of this manuscript? A great proportion of the results section is dedicated to the analysis of oscillations, while the main objective of the paper is on the role of calcium phosphate dissolution in the elevation of cytosolic calcium.
3) On the same note, several experimental and modeling studies have shown the importance of mitochondrial calcium oscillations in the sustainment and even regulation of cytosolic oscillations (e.g. Rizzuto et al. Nat Rev Mol Cell Biol. 2013, Wacquier et al. Sci. Rep. 2016, Qi et al. Sci. Rep. 2015, Pecze et al. J Biol Chem 2015). By assuming a constant reservoir of calcium in the mitochondria where calcium is continuously expelled into the cytosol such dependencies are greatly undermined.
4) Why only fluxes through the NCLX and the leak (Eqns. 12 and 13) have dependency on the IMM voltage and not the MCU (Eqn 11)? Experimental evidence suggests that the mitochondrial Ca^2+^ uptake is largely dependent on, and is driven by, the voltage across the IMM (e.g., Csordás, G. et al. Cell Metab. 2013). Additionally, several previous modeling studies on the mitochondrial calcium dynamics have accounted for this dependency for the flux through the MCU channels (e.g. Qi et al. Sci. Rep. 2015; Wacquier et al. Sci. Rep. 2016).
5) Why is the leak flux for the mitochondrial compartment (Eqn. 13) defined as an inward flux into the mitochondria? More importantly, by assuming sustained high concentrations of mitochondria during CSD, shouldn’t the direction of this flux be outward? Please clarify.
6) Please specify the inward or outward fluxes (i.e., positive and negative signs) in the balance equations and not in the definition of the flux itself, for example the negative sign in Eqns. 11, 13, 25, 29 and 30. Additionally, why does the flux through VOCC channels have a negative sign in Eqn. 25? Is this a typo?
7) According to numerous experimental and modeling studies, the open probability of the IP3R channels is dependent both on the level of calcium and IP3 in the cytosol. It seems that the IP3 dependency of the IP3R dynamics is not included in the model (Eqns. 20 and 21), even thought it is shown in the schematic of Fig. 1. Please clarify.
8) The parametric analysis is performed by scaling the influx into/outflux from the cytosol by a factor α ranging from [0.1, 10], and the leak fluxes are adjusted such that the initial concentrations remain as stated in Table 5.
a) First concern is that by changing the leaks, effectively the fluxes within, or across, compartments are re-adjusted and therefore the resulting changes cannot be attributed to the change in the specified flux.
b) The authors need to provide information, for instance in the form of histograms or similar approaches, that the levels of calcium in the compartments remain within physiological bounds upon rescaling of the fluxes.
c) If the parametric analysis is performed to analyze the effect of relevant fluxes on the time scale of calcium fluxes during CSD, why the mitochondrial calcium is kept at 0.1 µM and not at the elevated level.

Please refer to previous modeling studies for a more thorough local or global sensitivity analysis of the parameters.

9) What is the difference between panels of Fig. 5? It seems that the axes are the same in all panels. Please clarify.
10) According to the Fig. 3 legend, positive fluxes correspond to flow into the cytosol. Accordingly, in Fig. 7, the majority of simulations indicate that there is a net influx into the mitochondria (negative value), rather than outflux. Is the convention for the positive flux consistent across all figures?
11) In the third paragraph of Section 3.2, the authors provide a speculative explanation of why simulations in Fig. 3 predict a timescale of over 30 minutes to expel the absorbed calcium during CSD. Firstly, it should be noted that the conclusions are drawn based on concentrations and not based on the amount (moles) of calcium entering and exiting different compartments. Since compartments have different volumes, the explanation cannot be based on concentrations. Secondly, all these assumptions can be directly tested in the model by changing the volume of the extracellular compartment, buffering capacity of the cytosol and rate of uptake from the mitochondria. Thus, in my opinion the authors should expand their analysis/model to account for relevant observations during CSD instead of drawing conclusions based on assumptions.

Some additional comments:

1) In the first paragraph of the Introduction, is it stated that “*Cortical spreading depolarization (CSD), also known as cortical spreading depression …*”. Please note that spreading depolarization and cortical spreading depression are two separate phenomena with several distinct differences (please refer to Pietrobon et al. Nature Reviews Neuroscience 2014). Thus, these two terms should not be used synonymously.
2) Although the authors provide compelling evidence for the persistent vasoconstriction during the second phase of CSD as in numerous other studies in literature, there are also some studies (e.g. Brennan et al. J of Neurophys. 2007) that show sustained vasodilation after the passage of the CSD wave in some animal species. Perhaps commenting on these distinct vascular responses and the potential contribution of various vasoactive agents released during this phenomenon can be a relevant addition to the discussion.
3) Since elevation of the calcium in the mitochondria resulted in a substantial change in the level of calcium in the ER/SR, it would be more proper to incorporate store operated calcium (SOC) channels on the plasma membrane.
4) In the first paragraph of section 2, “*… generally parameterized in units of millimolar (µ-moles per liter) …”*, millimolar seems to be a typo 🡪 micromolar
5) Oscillations in Fig. 3 seem to show changes in the amplitude. Please double check if this is not because of time discretization used for the simulations or is not a solver issue.
6) Some of the dots in Fig. 7 are out of the axes. Either remove them or show another angle of the plot so that their locations are more visible.
7) Please add the description of all model parameters in the table. Also, please make sure that all parameter values are specified. For instance, I could not find the value of p2 in the table.
