# Supplementary material for: A mathematical model for persistent post-CSD vasoconstriction
Source: PLoS Comput Biol. 2020 Jul 15;16(7):e1007996. doi: 10.1371/journal.pcbi.1007996 (PMC7416967; doi:10.1371/journal.pcbi.1007996)
Supplement: Attachment — Submitted filename: plosreport.docx [file pcbi.1007996.s005.docx]

The manuscript deals with the dynamics of Ca^2+^ clearance from vascular smooth muscle cells in the brain after cortical spreading depression. It takes both mitochondrial and ER/SR Ca^2+^ concentration dynamics into account. The free mitochondrial Ca^2+^ concentration is assumed to be constant due to a quasi-static equilibrium of calcium phosphate cluster formation. With the assumptions of the model, the only remaining question is whether the mit->cyt flux or the cyt->ecs flux is rate limiting. The authors opt for the second choice. What is the experimental evidence supporting this choice? In general, the connection to experimental results is not well presented. Why are experimental data from published literature not included?

Minor topics

- The authors have chosen to include Ca^2+^ regulation of PLC. Is there experimental evidence that PLCδ or PLCη are present in these cells?
- Section 2, 2. Par “generally parameterized in units of millimolar (µ-moles per liter).” Milli or micro?
- Close to end of section 1, the singular is mitochondrion not mitochondria.
- Justify choices of models for IP3R, RyR, SERCA, MCU, references?
- It has been shown that slow mitochondrial efflux affects the fraction of Ca2+-inhibited IP3R between ER release spikes (Biophysical Journal Volume 77 July 1999 37–44). Is this also the case in the model presented here? Why?
