# Supplementary material for: A mathematical model for persistent post-CSD vasoconstriction
Source: PLoS Comput Biol. 2020 Jul 15;16(7):e1007996. doi: 10.1371/journal.pcbi.1007996 (PMC7416967; doi:10.1371/journal.pcbi.1007996)
Supplement: Attachment — Submitted filename: responseletter kcb_sx_hh.docx [file pcbi.1007996.s006.docx]

We would like to thank all referees for their very helpful comments and suggestions. We revised our paper to address all of them in detail. In what follows, we provide our responses to all individual comments and describe in detail specific changes in the revised manuscript made in response to the specific referees' comments and suggestions. Changes in our manuscript are indicated in Responses in blue letters.

______________________________________________ _____

**Responses to Referee #1**

Comment: *The manuscript by Xu et al. provides a compartmental model of a representative vascular smooth muscle cell to investigate the hypothesis, as well as implications of mitochondrial permeability transition pore (mPTP)-driven elevations of mitochondrial calcium concentration during the acute phase of cortical spreading depression (CSD) on the long-term vasoconstriction observed during the second phase of this phenomenon. The authors modeled the interaction between 4 Ca^2+^ pools, i.e., Cytosol, ER/SR, Mitochondria and the Extracellular space, by providing relative fluxes within and across compartments, and assuming fast buffering approximation for the cytosol and ER/SR. They assumed that the mitochondria exist at “quasi-steady” equilibrium at saturation and demonstrated that the slow release of calcium from this compartment to the cytosol can potentially explain long-term sustained elevations in the cytosol and the resultant vasoconstriction during CSD.*

*Although mathematical modeling of the vascular response during CSD is much needed to advance our understanding of the intricacies of this multi-mechanistic phenomenon, there are several concerns related to the study of Xu et al. that the authors should address.*

Response: We thank the reviewer for the evaluation of our paper and for the most useful suggestions that have significantly improved the paper.

Comment 1: *My main concern with the current study is that even though the main findings of the manuscript lies on the dynamics of calcium in the mitochondria and the calcium-phosphate dissolution/aggregation within this compartment (as is reflected in the title, abstract and discussion), the concentration of free mitochondria calcium is assumed to be constant throughout the long-term simulations, and none of the dynamics of calcium phosphates are included in the model. What is the basis of this assumption? Why did the authors opt not to provide calcium dynamics in the mitochondria to observe how long it takes for the calcium levels to reduce to physiological values?*

*If the model/study is specifically designed to investigate the role of mitochondria, in particular dynamics of mitochondrial calcium phosphate dissolution during CSD, it would be much more informative to provide a more detailed dynamics of this compartment (consistent with what is assumed for the other compartments of the model, such as ER/SR, Cytosol and the plasma membrane channels) and demonstrate the validity of these assumptions as well as the findings of the study. Similarly, in the abstract, the authors state “… we quantify a possible impact of calcium phosphate dissolution on the maintenance of F0F1-ATP synthase activity.” without providing any equations on the dynamics of proton balance across the mitochondrial inner membrane (IMM) and the ATP production for such quantification.*

Response: We thanks reviewer to point this problem out and great suggestion. After cortical spreading depolarization (CSD), huge amounts of calcium are absorbed in mitochondria and there are a lot of calcium-phosphate clusters have formed. When calcium and phosphate are at sufficiently high concentrations, they co-aggregate to form complexes of varying stoichiometry [56], first as prenucleation clusters, and then as stable nuclei.

The actual chemical pathways underlying these transformations are complex involving ATP and protons but rapid relative to the rate of exchange mechanisms present in the mitochondria membrane. We account for solubility in the mitochondria by invoking a quasi-steady state assumption on the concentration of free calcium under the rapid equilibration of dissolution and aggregation of calcium phosphate species. In this paper, "calcium-phosphate dissolution" mainly means the calcium clusters maintain an elevated constant calcium concentration in the mitochondria after CSD. Based on this assumption, we examine the implications of this persistent calcium elevation on cellular calcium dynamics and hour-long disruption in vascular function. Also, the lower bound of mitochondria elevation calcium concentration is consistent with the previous work.

Regarding the model of formation and dissolution of the cluster, we plan to do further careful study in the a separate paper. At same time, in order to remove the confusion, we change the title to be "A mathematical model for persistent post-CSD vasoconstriction"

Comment 2: *Although it is interesting to show the presence of oscillations in the cytosolic calcium concentrations during elevated levels of mitochondria calcium, how are these oscillations (and the analysis of the effect of different fluxes on their sustainment) relevant to the main objective of this manuscript? A great proportion of the results section is dedicated to the analysis of oscillations, while the main objective of the paper is on the role of calcium phosphate dissolution in the elevation of cytosolic calcium.*

Response: We are very sorry for the confusion due to the title. The main object of this paper is to analyze the effect of elevated constant calcium in mitochondria, which is maintained by calcium-phosphate dissolution, on the cytosolic calcium dynamics. The oscillations are relevant insomuch as they determine the macroscopic physiological observable of vasoconstriction that is seen in experiments.

The key assumption is that after CSD, the calcium inside mitochondria is at saturation point. The model and simulation results predict that in order to get an hour-long disruption, the saturation point of mitochondria should above 0.25$\mu M$. But the dynamics of calcium cluster dissolution and corresponding ATP and pH value changes are still not clear both experimentally and theoretically. We will collaborate with Kevin C. Brennan to get do further experimental study and then establish the mathematical model for the dissolution and depletion of calcium cluster inside the mitochondria.

Comment 3: *On the same note, several experimental and modeling studies have shown the importance of mitochondrial calcium oscillations in the sustainment and even regulation of cytosolic oscillations (e.g. Rizzuto et al. Nat Rev Mol Cell Biol. 2013, Wacquier et al. Sci. Rep. 2016, Qi et al. Sci. Rep. 2015, Pecze et al. J Biol Chem 2015). By assuming a constant reservoir of calcium in the mitochondria where calcium is continuously expelled into the cytosol such dependencies are greatly undermined*.

Response: The referee is correct that mitochondria play an important role for calcium oscillation. But the phenotype we describe is the post-acute dynamics, when the calcium concentration is at saturation point due to the formation of cluster. Also the calcium entry in CSD is HUGE – much larger than what is described in other work – thus enough loading for a long efflux period. Current work shows that the saturation concentration (or the calcium cluster inside the mitochondria) also plays important roles for cytosolic calcium oscillation.

Comment 4: *Why only fluxes through the NCLX and the leak (Eqns. 12 and 13) have dependency on the IMM voltage and not the MCU (Eqn 11)? Experimental evidence suggests that the mitochondrial Ca^2+^ uptake is largely dependent on, and is driven by, the voltage across the IMM (e.g., Csordás, G. et al. Cell Metab. 2013). Additionally, several previous modeling studies on the mitochondrial calcium dynamics have accounted for this dependency for the flux through the MCU channels (e.g. Qi et al. Sci. Rep. 2015; Wacquier et al. Sci. Rep. 2016).*

Response: Apologies, this is a typo. MCU is dependent on the membrane potential. Actually here we inherited Wacquier’s model. The corresponding parameters are also added to Table 2, and are in our online code.

Comment 5: *Why is the leak flux for the mitochondrial compartment (Eqn. 13) defined as an inward flux into the mitochondria? More importantly, by assuming sustained high concentrations of mitochondria during CSD, shouldn’t the direction of this flux be outward? Please clarify.*

Response: It is a bi-direction flux depending on the balance of membrane potential and Nernst potential, which could be seen from Fig. 3. When the concentration in cytosol is lower that mitochondria one (the beginning period), $J_{mit\to cyt}$ is positive, i.e, calcium leaks from mitochondria to cytosol. Later on, when the cytosolic concentration is higher that mitochondria one, $J_{mit\to cyt}$ is negative, i.e calcium leaks from mitochondria to cytosol.

Comment 6: *Please specify the inward or outward fluxes (i.e., positive and negative signs) in the balance equations and not in the definition of the flux itself, for example the negative sign in Eqns. 11, 13, 25, 29 and 30. Additionally, why does the flux through VOCC channels have a negative sign in Eqn. 25? Is this a typo?*

Response: Thanks referee’s suggestion. We added the explanations to each flux in order to clarify the direction of _ux. Here, since we mainly focus on the cytosolic concentration dynamics, we assume all flux J " to be oriented so that positive J is relative to positive changes in cytosolic calcium concentration.

Additionally, all J fluxes are scaled to the cytosolic volume." About the VOCC, that was because we use $-(\phi_{\mathrm{ecs}}-E_{ca,ecs})$ and $\phi_{\mathrm{ecs}}=-54$. So the totoal term is positive. But to obviate misunderstanding, in the revised version, we use

$$J_{\mathrm{vocc}}=Q_{vocc} \bar{d_{l}} \bar{f}_{l}(E_{\mathrm{Ca}},ecs-\phi_{\mathrm{ecs}})$$

Comment 7: *According to numerous experimental and modeling studies, the open probability of the IP3R channels is dependent both on the level of calcium and IP3 in the cytosol. It seems that the IP3 dependency of the IP3R dynamics is not included in the model (Eqns. 20 and 21), even thought it is shown in the schematic of Fig. 1. Please clarify.*

Response: In our model, we do consider the IP3R dynamics. In equation (21) (orignal version equation20), the term $(X_{10}^{4}+4X_{10}^{3}(1-X_{10}))$ is the signal channel open probability of IP3R, which depends onthe binding states. IP 3R is treated as have four binding states X00; X10; X01; X11. And the dynamics of those states are decribed in equation (20) (orignal version equation 21).

Comment 8: *The parametric analysis is performed by scaling the influx into/outflux from the cytosol by a factor α ranging from [0.1, 10], and the leak fluxes are adjusted such that the initial concentrations remain as stated in Table 5.*

a) *First concern is that by changing the leaks, effectively the fluxes within, or across, compartments are re-adjusted and therefore the resulting changes cannot be attributed to the change in the specified flux.*
b) *The authors need to provide information, for instance in the form of histograms or similar approaches, that the levels of calcium in the compartments remain within physiological bounds upon rescaling of the fluxes.*
c) *If the parametric analysis is performed to analyze the effect of relevant fluxes on the time scale of calcium fluxes during CSD, why the mitochondria calcium is kept at 0.1 µM and not at the elevated level.*

*Please refer to previous modeling studies for a more thorough local or global sensitivity analysis of the parameters.*

Response: a) Here we would like to study the parametric determinants of calcium dynamics in our model by looking at the impact of relevant fluxes on the time scale of different compartments (cytosol, mitochondria and ER). By choosing these transformations, it does not change the steady state.

b)The histogram of [Ca2+]cyt is shown as follows. It illustrates that the concentration is in the physiological region.

c) The values in Table 5 are used to constrain the free parameters in the model. During the parametric analysis, the concentration in mitochondria is the elevated value $0.25\mu M.$ In the revised version, we clarify this: "We study the parametric determinants of calcium dynamics in our model by looking at the impact of relevant fluxes on the time scale of calcium fluxes with fixed elevated mitochondria concentration $0.25\mu M$ via

$$J_{\left( \cdot\right)\to cyt}\to\alpha_{(\cdot)}J_{\left( \cdot\right)\to cyt}$$

where $\alpha_{(\cdot)}$is the timescale factor which mean how fast of each compartment achieves steady states. "

Comment 9: *What is the difference between panels of Fig. 5? It seems that the axes are the same in all panels. Please clarify.*

Response: Fig. 5 is the same figure with different rotation angle to illustrate the elect of flux re-scaling on the oscillation. It is updated.

Comment 10: *According to the Fig. 3 legend, positive fluxes correspond to flow into the cytosol. Accordingly, in Fig. 7, the majority of simulations indicate that there is a net influx into the mitochondria (negative value), rather than outflux. Is the convention for the positive flux consistent across all figures?*

Response: That is because of the confusion of flux scale and visual angle. The convention for positive flux is consistent across all figures, i.e. from other compartments to cytosol. In the revised version, Figure 7 clearly illustrates that most values (80%) of $J_{mit\to cyt}$ is positive, i.e., from mitochondria to ocytosol, under different timescale $\alpha_{mit,ecs.cyt}$ The results in Figure 3 are the results when the elevated concentration of mitochondria is $0.25\mu M$ and $\alpha_{mit,ecs.cyt}=1$.

Comment 11: *In the third paragraph of Section 3.2, the authors provide a speculative explanation of why simulations in Fig. 3 predict a timescale of over 30 minutes to expel the absorbed calcium during CSD. Firstly, it should be noted that the conclusions are drawn based on concentrations and not based on the amount (moles) of calcium entering and exiting different compartments. Since compartments have different volumes, the explanation cannot be based on concentrations. Secondly, all these assumptions can be directly tested in the model by changing the volume of the extracellular compartment, buffering capacity of the cytosol and rate of uptake from the mitochondria. Thus, in my opinion the authors should expand their analysis/model to account for relevant observations during CSD instead of drawing conclusions based on assumptions.*

Response: Figure 4 has been updated by using the unit mol, i.e. the amount of calcium entering the esc from cytosol and entering cytosol from mitochondria as following in Section 3.2..

The different time scale is based on the different mechanism on Mitochondria flux, cell membrane flux and ER flux. By using that $\alpha_{i}$, the steady state of each compartments does not change.

Comment 12: *Some additional comments:*

1) *In the first paragraph of the Introduction, is it stated that “Cortical spreading depolarization (CSD), also known as cortical spreading depression …”. Please note that spreading depolarization and cortical spreading depression are two separate phenomena with several distinct differences (please refer to Pietrobon et al. Nature Reviews Neuroscience 2014). Thus, these two terms should not be used synonymously.*
2) *Although the authors provide compelling evidence for the persistent vasoconstriction during the second phase of CSD as in numerous other studies in literature, there are also some studies (e.g. Brennan et al. J of Neurophys. 2007) that show sustained vasodilation after the passage of the CSD wave in some animal species. Perhaps commenting on these distinct vascular responses and the potential contribution of various vasoactive agents released during this phenomenon can be a relevant addition to the discussion.*
3) *Since elevation of the calcium in the mitochondria resulted in a substantial change in the level of calcium in the ER/SR, it would be more proper to incorporate store operated calcium (SOC) channels on the plasma membrane.*
4) *In the first paragraph of section 2, “… generally parameterized in units of millimolar (µ-moles per liter) …”, millimolar seems to be a typo 🡪 micromolar*
5) *Oscillations in Fig. 3 seem to show changes in the amplitude. Please double check if this is not because of time discretization used for the simulations or is not a solver issue.*
6) *Some of the dots in Fig. 7 are out of the axes. Either remove them or show another angle of the plot so that their locations are more visible.*
7) *Please add the description of all model parameters in the table. Also, please make sure that all parameter values are specified. For instance, I could not find the value of p2 in the table.*

Response: 1) Thanks for clarifying. It is changed to be "Cortical spreading depression (CSD)".

2) There are indeed species differences in the vascular response to CSD. However these differences mainly pertain to the early vascular response, and not the long lasting post-csd vasoconstriction, which is present in all models tested including humans. In all species, there are up to four phases of vascular diameter change – an inconsistently seen dilation/hyperperfusion just before wave onset, a constriction/hypoperfusion at the onset of the wave, a dilation/hyperperfusion later in the csd wave, and a post-csd constriction/hypoperfusion. The CSD-associated constriction/hypoperfusion and dilation/hyperperfusion vary most between species (e.g. constriction phase is much larger in mice than rats; dilation phase much larger in rats than mice) but again the post-CSD changes we are modeling are quite consistent. This is well detailed in the Ayata and Lauritzen 2014 review. Because the phenomenon we are examining is consistent, we favor holding off on additional explanation in the discussion, as it might appear superfluous, but are open to adding this explanation if preferred by the reviewers.

3) In the model, the calcium dependent dynamic buffering (Eqs. 16-17) is considered to ensure that

the ER concentration is in the reasonable region. The concentration in Figure 3 is in the range of

previous work like [Johny Biophiscal 2017] and [Marcucci Plos one 2018].

4) It is corrected.

5) The figure is updated now.

6) The figure is updated now.

7) The description of all model parameters are added.

______________________________________________ _____

**Responses to Referee #2**

Comment: *A primary goal of this paper is to develop a computational model to help explain mechanisms underlying neurovascular malfunction following cortical spreading depression. This is an interesting issue and the modeling is novel because, as the authors point out, it involves time scales not considered in previous models for CSD. However, I have several major concerns that are outlined below.*

Response: We thank the reviewer for the positive evaluation of our paper and for the most useful suggestions that have significantly improved the paper.

Comment 1: *The authors claim in the Discussion that they “demonstrated macroscopic vascular behavior consistent with the hour-long disruption in neurovascular coupling following cortical spreading depolarization.” I interpret this to mean that there is some variable corresponding to vascular behavior (say vessel diameter), which is in some constrictive state following CSD and then returns to a resting state after about one hour. I don’t see where they show this. The only place in the results section where the vascular activity is described is Figure 4; however, the muscle tension does not return to a resting state after one hour. In fact, neither do the other variables.*

Response: In the model, we modelled vasoconstriction and dilation indirectly through a four-state model of calcium-dependent actin/myosin dynamics Eq.(5). And the tension generated by smooth muscle cell (Fr) is a linear function of attached phoshorylated cross-brides(AMp) and attached dephosphorylated non-cycling cross bridge (AM). The simulation result in Fig. 4 third column shown the oscillation of the tension, which illustrates the vasoconstriction. Yes, in the real experiment, the calcium concentration and diameter come back to normal values after hour-long time. However, the cluster depletion and ATP production inside the mitochondria are very complicate and not clear after the formation of calcium cluster. So here we assume that there are enough calcium clusters and ATP inside the mitochondria to focus on the effect of elevated calcium in mitochondria to the cytosolic dynamic. The detailed cluster deletion and concentration of phosphate will be studied both in experimentally and theoretically by collaboration with K. C. Brennan group.

Comment 2: *The authors need to explain the actin/myosin model (5). What do the variables correspond to and what values correspond to a constrictive or resting state?*

Response: Thanks for pointing it out. We have added the explanations to variables, reference. The resting state is shown in Table 5.

Comment 3: *The authors seem to justify their results by stating that “the dynamics in this set of simulate are consistent with the observed vasoactivity of Figure 4A in Chang et al.” They need to explain this claim in some detail. It is not at all obvious how the simulations in this paper correspond to what is shown in Chang et al.*

Response: In Chang et al Fig 4 we observed an approximately hour long constriction in cortical surface arteries after CSD, with superimposed oscillations. This is now replotted in Figure 4. The fact that our model generates a predicted constriction, including oscillations, in a time scale on the order of magnitude of those observed after CSD is in our estimation quite a promising result. As the reviewer points out, thus far there has been no modeling of CSD associated events on this time scale, despite the fact that the ‘aftermath’ of CSD is much longer than the wave, and may have significant effects on vascular and network function (e.g. Chang et al 2010, Theriot et al J Neurosci 2013, Sawant-Pokam et al Cereb Cortex 2016). Though our modeled constriction is shorter at ~1/2 hour, we suspect, as detailed in the Discussion, that because of simplifying assumptions (lack of calcium re-entry from VOCC, lack of mitochondria ATP rundown), we are underestimating the actual duration.

Comment 4: *The authors make many, many assumptions, which raise concerns. For example:*

*a) The mitochondria membrane potential ΔΨm is considered to be fixed. However, a large increase in mitochondrial calcium may lead to a. large change in ΔΨm and this would impact all aspects of mitochondrial dynamics.*

*b) It is assumed that hat large quantities of calcium have entered the mitochondria during the acute phase of CSD, through the mPTP. Isn’t opening of the mPTP associated with cell death?*

*c) The caption of Table 5 states that “Steady state free ionic calcium concentrations*

*enforced in all of the model simulations.” But the figures show changes in these concentrations.*

*d) I don’t understand the statement: “In these simulations the stabilizing leak fluxes are calibrated in order to maintain the original unperturbed steady state.”*

*e) The role of ATP is confusing. At one point, the authors state: “we assume that ATP production meets the needs of this system.” However, ATP production is described in some detail in, for example, Sections 2.5 and 2.6. Why is the ATP production rate proportional to*$J_{mit\to cyt}$*? It is possible that the large increase in mitochondria calcium leads to a sharp decrease in ATP production due to changes in ΔΨm described above.*

*Response:*

a) After the acute phase CSD, those large amount of calcium is saved as the form of clusters, and the free calcium concentration is at the saturation point. Here we actually assume that transformation between clusters and free ion is fast enough such that the mitochondria is at quasi-steady state. Combining with the extreme fast $H^{+}$ pump, the membrane potential could be a constant.
b) MPTP opening was once thought to be exclusively associated with cell death but it is now known to be potentially reversible, in particular during CSD – Piilgaard and Lauritzen (JCBFM 2011) show MPTP activation during CSD that does not result in the death of the cell and is thus reversible. More broadly the concept of transient MPTP opening, without cell death, has become established in the neuroscience field (e.g. Agarwal et al Bergles, Neuron 2017).
c) Values in Table 5 are the values of cell under normal circumstance and are used to constrain the dimensionality of free parameters in the model. After those free parameters are fixed, different elevated mitochondria concentration is studied to generate Figure 2. For Figures 3-7, the concentration of mitochondria is fixed as $0.25\mu M$ which is away from the normal circumstances.
d) Since in the model, the leak rates are some free parameters. We first constrained these parameters by ensure the steady-state of cell under normal circumstance shown in Table 5. And then only varied the mitochondria concentration.
e) Similar as response (a), due to the quasi-steady state of mitochondria and saturation of calcium, the membrane potential is constant. Then according to Wacquier Scientific Report 2015 eqs. (5) and (7), we could assume that the production of ATP linearly depends on $J_{mit\to cyt}$. Even though, we assume that the production of ATP is enough for simplicity, but $J_{mit\to cyt}$ could tell us how much ATP is produced roughly.

______________________________________________ _____

**Responses to Referee #3**

Comment 1: *The manuscript deals with the dynamics of Ca2+ clearance from vascular smooth muscle cells in the brain after cortical spreading depression. It takes both mitochondrial and ER/SR Ca2+ concentration dynamics into account. The free mitochondrial Ca2+ concentration is assumed to be constant due to a quasi-static equilibrium of calcium phosphate cluster formation. With the assumptions of the model, the only remaining question is whether the mit->cyt flux or the cyt->ecs flux is rate limiting. The authors opt for the second choice. What is the experimental evidence supporting this choice? In general, the connection to experimental results is not well presented. Why are experimental data from published literature not included?*

Response: We now include a re-plotted vascular diameter plot from Chang et al 2010, showing the key predicted vascular phenotypes of 1. Sustained constriction, 2. Oscillations superimposed on the constriction. Unfortunately, other sources of physiological data, e.g. smooth muscle cell calcium levels during and after CSD, are not currently obtainable – this is actually a major motivation for our in silico experiments. However we have an ongoing collaboration with the laboratory of K.C. Brennan – we hope that in the near future, we will be able to present higher resolution diameter and flow measurements from cortical vessels, and possibly smooth muscle cell calcium measurements, as the technology improves.

Comment 2: *The authors have chosen to include Ca2+ regulation of PLC. Is there experimental evidence that* $PLC_{\delta}$ *or* $PLC_{\eta}$*are present in these cells?*

Response: Yes, based on the work "Rebecchi, Mario J., and Srinivas N. Pentyala. "Structure, function, and control of phosphoinositide-specific phospholipase C." Physiological reviews 80.4 (2000): 1291-1335." and "Rebecchi, M.J., Pentyala, S.N., 2000. Structure, function, and control of phosphoinositidespecific phospholipase C. Physiol. Rev. 80, 1291-1335", they state that $\mathrm{PLC}_{\delta}$ are present in smooth muscle cells.

Comment 3: *Section 2, 2. Par “generally parameterized in units of millimolar (µ-moles per liter).” Milli or micro?*

Response: Thanks for careful reading. It is a typo. It should be micromolar.

Comment 4: *Close to end of section 1, the singular is mitochondrion not mitochondria.*

Response: Thanks. It is corrected.

Comment 5: *Justify choices of models for IP3R, RyR, SERCA, MCU, references?*

Response: The references for IP3R, RyR, SERCA, MCU are added. For the IP3R and RyR channels, the open probabilities are affected by cytosolic calcium concentration directly and the receptors have different states when calcium binds to the receptors. So the multi-state model is used. About the SERCA, the pumping capacity of SERCA pump is determined by the total concentration of pump protein. It is also affected by both cytosolic and ER calcium concentrations. So the [Higgins et al. 2006] model is used. For the MCU flux, it is membrane potential dependent channel, so the model in [Wacquier et al.2016] is inherited.

Comment 6: *It has been shown that slow mitochondrial efflux affects the fraction of Ca2+-inhibited IP3R between ER release spikes (Biophysical Journal Volume 77 July 1999 37–44). Is this also the case in the model presented here? Why?*

Response: Yes. That is because the IP3R open probability depends on the cytosol calcium concentration, and the mitochondrial efflux change the concentration of calcium Actually currently model is more complex that the four-states model is used to model the IP3R activation. Johny et.al Biophysical Journal 2015, Figure 2 (see below) has confirmed the relation between cytosol calcium and IP3R open probability and validated with the experimental observation.

______________________________________________ _____

**Responses to Referee #4**

Comment: *The paper deals with the modelling of the cortical spreading depolarization (CSD), a relatively slow wave propagating in cortical brain tissue during migraines, strokes etc. As such it has the potential to provide a good contribution to the literature if the following points below are addressed carefully. The experimental results the model is compared to are those in Chang et al, Figure 4A.*

Response: We thank the reviewer for the positive evaluation of our paper and for the most useful suggestions that have significantly improved the paper.

Comment 1: *Please provide a clear explanation of the experimental results that you are comparing against with your model. Please include Figure 4A from Chang et al, preferably early on, for the sake of clarity.*

Response: We now include a re-plotted trace of vascular diameter from Chang et al Fig 4, and emphasize the key phenotypes that our model replicates: 1. Sustained constriction; 2. Oscillations superimposed on the constriction.

Comment 2: *Section 2: Please provide a clearer explanation of the modelling modules you have introduced for the cytosol, mitochondria and the Endoplasmic Reticulum. Why have you chosen these models vs alternatives? What are the key assumptions? Also, more specifically: a. in Section 2.1 the variables AP, AMp, MP and the parameters K1, K2, K3 etc in the equation system (5) are not defined. Also it is not mentioned if/where the model has been developed before. I presume in Yang et al [71]?*

*b. In equation (11) why do you model Jmcu in this way? What is the biophysical process justifying this choice?*

*c. System (21): why do you choose the model by Swaminathan et al [60] vs other established IPR models?*

Response: For the model in ER, we considered the buffer effect and limited total binding protein. For the IP3R and RyR channels, the open probabilities are affected by cytosolic calcium concentration directly and the receptors have di_erent states when calcium binds to the receptors. So the multi-state model is used. About the SERCA, the pumping capacity of SERCA pump is determined by the total concentration of pump protein. It is also affected by both cytosolic and ER calcium concentrations. So the [Higgins et al. 2006] model is used. For the Mitochondria model flux, they are membrane potential dependent channel, so the models in [Wacquier et al. 2016] are inherited.

a) The explanations to the variables and parameters are added. Also the reference is added.

b) Here we used the model in [Wacquier et al. 2016] which is based on Monod-Wyman-Changeux formalism for allosteric enzymes and further assumes that calcium cannot bind to transporter when subunits are in the so-called "tense" form since the flux depends on mitochondria membrane potential and cytosolic calcium concentration. In Wacquier’s paper, it is illustrated that the model is consistent with the experimental results.

c) For the Ip3R channels, we use the same assumption as the RyR channels. There are four binding states and the binding dynamics should be affected by both cytosolic calcium concentration and IP3 concentration. Also, in [Johny et al. 2016], authors illustrate that it is consistent with experimental observations and [Kapela et al.2008] results.

Comment 3: *It is not clear which part of the modelling is new and which has been developed in previous work. The novelty of the work should be clearly established.*

Response: Thanks for pointing it out. In the revised version, all the references for the existing models are added.

Comment 4: *Section 3: The comparison of the simulations with the experimental results is not convincing. In Figure 4A of Chang et al the variables plotted and tracked are mostly different from the variables of the ODE model used in the current paper. One variable that should be definitely included, or at least discussed in detail when the modelling variables are introduced, is the Pial Artery Diameter which is constricting as the CSD passes.*

Response: This is included as requested – please see also responses above.

Comment 5: *An “hour-long” vasoconstriction effect is reported but the simulations provided run only up to 30 minutes. What happens afterwards? Can you provide some pictures over the duration of an hour?*

Response: The 1H simulation results are presented as follows: In the real experiment, the calcium concentration and diameter come back to normal values after hour-long time. However, the cluster depletion and ATP production inside the mitochondria are very complicate and not clear after the formation of calcium cluster. So here we assume that there are enough calcium clusters and ATP inside the mitochondria to focus on the effect of elevated calcium in mitochondria to the cytosolic dynamic. The detailed cluster deletion and concentration of phosphate will be studied both in experimentally and theoretically by collaboration with K. C. Brennan group.

Comment 6: *Figure 2: Are there perhaps more bifurcations beyond the Hopf bifurcation?*

Response: It is not clear that more bifurcations exist beyond the Hopf bifurcation discussed in our manuscript. We plan to carry out a more detailed analysis in a follow up study.

Comment 7: *Figure 2: Can you explain why the Hopf bifurcation is observed in all variables at the same value of calcium?*

Response: The Hopf bifurcation observed is due the IP3 dynamics when Calcium exceeds a critical value.

Comment 8: *Figure 2 caption: Not sure why the sentence “Fr is proportional to the constrictive force….” Is included here. If you are leaving it here please connect better to the context.*

Response: Thanks for pointing this confusing sentence out. It is deleted in the updated version.

Comment 9: *CSD is a wave but only ODEs are used in the model. Spatial dependence is needed for a wave to propagate. The authors should clearly explain why they have neglected spatial effects, dealing only with oscillations and not waves.*

Response: In the "Assumptions, limitations and alternative mechanisms" section, we add the following statements

"Finally, in the current work, we mainly focus on proposing a simple model for the hour-long oscillation in the one cell after the acute phase CSD. In order to consider the wave propagation along the tissue, the spacial variation need to be taken into consideration. A partial differential equations model like in [Kennya et al.2019] could help to connect the macro scale wave propagation with micro cytosol calcium oscillation."

Comment 10: *p.10 “Results” section. Please explain what DSP.jl is doing. Is it a bifurcation tracking module?*

Response: In the revised version, it is clarified as follows: " We ascertained the frequency of any oscillations using DSP.jl, (Github: JuliaDSP/DSP.jl) which is a common digital signal processing package for Periodogram estimation."

Comment 11*: p. 11 Why do you introduce αi? Not clear.*

Response: In the revised version we added the meaning of _i and explain the purpose as follows:

“We study the parametric determinants of calcium dynamics in our model by looking at the impact of relevant fluxes on the time scale of calcium fluxes with fixed elevated mitochondria concentration $0.25\mu M$ via

$$J_{\left( \cdot\right)\to cyt}\to\alpha_{(\cdot)}J_{\left( \cdot\right)\to cyt}$$

where $\alpha_{(\cdot)}$is the timescale factor which mean how fast of each compartment achieves steady states. "

______________________________________________ _____

**Responses to Referee #5**

Comment: *Summary: This manuscript assembles a computational model to explain elevated cytosolic calcium levels and clearance in vascular smooth muscle cells (VSMCs) of the cerebral vasculature after a cortical spreading depolarization (CSD) event. This model describes calcium transport through the cellular membrane, between the cytosol and the endoplasmic reticulum, and between the cytosol and the mitochondrial matrix. This model is able to describe the hour long timescale for cytosolic calcium to return to normal after a CSD event.*

Response: We thank the reviewer for the positive evaluation of our paper and for the most useful suggestions that have significantly improved the paper.

Comment 1: *Overall Comments: The authors have built a simplified model to represent the calcium levels in VSMCs after CSD and have clearly described their model in the text. They have also effectively varied parameters to try to understand the important parts of the model. However, no experimental data is shown and in none of the figures the actual rise and decay of calcium over the post CSD time period is shown. Parameters are clearly presented along with their sources which enables reproducibility however searching for the code under the username joshchang on github did not yield the code for inspection.*

Response: The experimental data from Chang Figure 4A is included now. The code now is available at github:xsxztr/xsxztr-Post-CSD-code-with-Joshua-C.-Chang.

Comment 2: *Specific Comments:*

*1) No data is represented to challenge the results of the model.*

*2) Make sure that code is available on github at least and it is suggested to take advantage of the code check that is an option with PLoS Comput Biol.*

*3) It is stated that in figure 2 the net movement of the calcium is from the mitochondria to the cytosol to the extracellular space however the net flux frommitochondria to cytosol in figure 2 is essentially zero at the resolution of the figure.*

*4) The simulations of figures 3 and 4 show some variability with no apparent pattern. Why is this the case if you simply have a set of ODEs modeled here?*

*5) The authors have made clear that local regulatory mechanisms have been omitted*

*in this model. However in the discussion the interaction between these Ca2+ transients and those generated by the myogenic response or shear mediated*

*dilation should be discussed.*

Response: 1)-2) The code and data are available for checking at github:xsxztr/xsxztr-Post-CSD-codewith-Joshua-C.-Chang.

3) The flux $J_{mit\to cyt}$in Figure 3 is oscillated around 0.5 to -0.5 (which actually is greater than -0.5). But the total integration of this flux with respect to time in Figure 4b illustrate that the calcium is leaked from mitchondria to cytosol.

4) The figures are updated and now the patterns are apparent.

5)This is indeed an important consideration, which we place in the category of potentially important factors that we have chosen not to model. We have added the following text to the discussion:

‘We have also not considered pressure- and flow-mediated changes in smooth muscle cell activity, which can result in both increases and decreases in cytosolic calcium.”

Comment 3*: Specific Minor comments*

*1) Figure 4 shows the clearance of Ca2+ in panel 1 and the release of Ca2+ from the mitochondria in panel 2 but does not resolve the time course of Ca2+ in the cytosol over the 30 minute timescale depicted.*

*2) Figure 5 is not really clear. Suggest one 3D view with 3 views comparing each pair of parameters (top, front and side).*

*3) On page 15 the authors suggest that cell-specific measurements would be beneficial for the identification of this model. Can the authors suggest what measures would have the most utility to advance this work?*

*4) Mitochondrial permeability transition pore opening leads to mitochondrial dysfunction and cell death. It is assumed in this model that the VSMCs recover. Is this tractable?*

Response: 1) The 1H simulation results are presented as follows: In the real experiment, the calcium concentration and diameter come back to normal values after hour-long time. However, the cluster depletion and ATP production inside the mitochondria are very complicate and not clear after the formation of calcium cluster. So here we assume that there are enough calcium clusters and ATP inside the mitochondria to focus on the effect of elevated calcium in mitochondria to the cytosolic dynamic. The detailed cluster deletion and concentration of phosphate will be studied both in experimentally and theoretically by collaboration with K. C. Brennan group.

2) The figure is updated.

3) The most useful single cell measurements for our purposes would be, in order of priority: 1. Vascular smooth muscle calcium; 2. Smooth muscle cell membrane potential as a proxy for smooth muscle calcium; 3. Neuronal calcium, glutamate, and/or membrane potential, as these allow us to follow the progress of CSD. The latter recordings are all routinely available in the Brennan lab; however the first two are not yet available. We are optimistic that these techniques will be deployable in the near future.

4) Please see answer above – recent advances show that MPTP opening without cell death occurs, including with CSD, and that transient MPTP opening appears to be a regulated biological process.
